# Hippocampal Feedforward Inhibition Focuses Excitatory Synaptic Signals into Distinct Dendritic Compartments

**DOI:** 10.1371/journal.pone.0080984

**Published:** 2013-11-11

**Authors:** Silvia Willadt, Markus Nenniger, Kaspar E. Vogt

**Affiliations:** Neurobiology/Pharmacology, Biozentrum, University of Basel, Basel, Switzerland; University of Exeter, United Kingdom

## Abstract

Feedforward inhibition controls the time window for synaptic integration and ensures temporal precision in cortical circuits. There is little information whether feedforward inhibition affects neurons uniformly, or whether it contributes to computational refinement within the dendritic tree. Here we demonstrate that feedforward inhibition crucially shapes the integration of synaptic signals in pyramidal cell dendrites. Using voltage-sensitive dye imaging we studied the transmembrane voltage patterns in CA1 pyramidal neurons after Schaffer collateral stimulation in acute brain slices from mice. We observed a high degree of variability in the excitation-inhibition ratio between different branches of the dendritic tree. Many dendritic segments showed no depolarizing signal at all, especially the basal dendrites that received predominantly inhibitory signals. Application of the GABA_A_ receptor antagonist bicuculline resulted in the spread of depolarizing signals throughout the dendritic tree. Tetanic stimulation of Schaffer collateral inputs induced significant alterations in the patterns of excitation/inhibition, indicating that they are modified by synaptic plasticity. In summary, we show that feedforward inhibition restricts the occurrence of depolarizing signals within the dendritic tree of CA1 pyramidal neurons and thus refines signal integration spatially.

## Introduction

Under physiological conditions principal cells in the central nervous system receive biphasic innervation patterns composed of excitatory postsynaptic potentials (EPSPs) from direct excitatory inputs and inhibitory postsynaptic potentials (IPSPs) from interneurons, activated by collaterals of the excitatory input [1,2]. The interval between the excitatory and inhibitory phases in such feedforward inhibition is short, in the order of a few milliseconds. This tight temporal coupling results in a significantly faster repolarisation than passive membrane properties would produce. In the hippocampus, Pouille and Scanziani [3] have shown that feedforward inhibition thus controls the time window for synaptic integration in pyramidal neurons, ensuring temporal precision in the hippocampal CA1 area. 

While the temporal characteristics and impact of feedforward inhibition are well understood, we know much less about its spatial properties. Synaptic integration takes place in the geometrically complex dendritic tree, which receives synaptic contacts over its entire surface. Synapses are not randomly distributed, but show remarkable specificity in their localisation. Both excitatory and in particular inhibitory synapses show input-specific preference for particular compartments. In the hippocampus, excitatory inputs arrive in a layer-specific organization with entorhinal input arriving at the most distal dendritic locations in stratum lacunosum moleculare and Schaffer collaterals (SCs) from the CA3 area arriving more proximally [4]. SC synapses themselves also show spatial organization, with inputs originating close to the hilus terminating in the apical stratum radiatum and inputs from cells close to CA1 forming synapses predominantly on the proximal apical dendrite in the stratum radiatum and onto basal dendrites in the stratum pyramidale/oriens [5]. Even more specific is the spatial distribution of synapses from different interneuron types, which has been used for their classification [1,6]. The relative location of excitatory and inhibitory synapses will significantly affect how these signals will interact [7].

We have recently shown that small inhibitory synaptic signals can be studied using voltage sensitive dye (VSD) imaging at sufficient resolution to allow the study of their dendritic integration within a single CA1 pyramidal neuron undisturbed by whole-cell recordings [8].

Here we study the effect of feedforward inhibition on dendritic signal integration using VSD imaging. Dye-loaded cells were imaged after SC stimulation and the voltage transients in different dendritic subcompartments were then analysed. 

We demonstrate a critical influence of γ-aminobutyric acid receptor- (GABA_A_R-) mediated inhibition on the subcellular membrane potential patterns in CA1 pyramidal neurons.

## Materials and Methods

### Brain slice preparation

All experiments were approved by Basel cantonal veterinary authorities. Recordings were performed in 300 µm thick brain slices of heterozygous knock-in mice P21 to P32 expressing GFP from the GAD67 gene locus [9]. After deep isoflurane anaesthesia mice were decapitated and transversal hippocampal slices were cut using a vibrating microtome (VT1200S, Leica, Switzerland). Slicing was performed in ice-cold solution containing (in mM) NaCl 87, Sucrose 75, Glucose 25, NaHCO_3_ 25, MgCl_2_ 7, KCl 2.5, NaH_2_PO_4_ 1.25, CaCl_2_ 0.5, equilibrated with 95% O_2_ and 5% CO_2_. After cutting, slices were incubated at 35°C for 30 min in artificial cerebrospinal fluid (ACSF), also used as extracellular solution for the experiments. This solution contained (in mM): NaCl 125, NaHCO_3_ 26, NaH_2_PO_4_*H_2_O 1.25, KCl 2.5, MgSO_4_ 1.0, CaCl 2.5; 310 mOsmol and pH 7.4 when bubbled with a gas mixture containing 95% O_2_, 5% CO_2_.

### Neuronal loading

CA1 pyramidal cells were loaded with the VSD JPW-1114 (0.2-0.5 mg/ml, Molecular Probes-Invitrogen) as described previously in detail [8,10].

While staining cells with VSD, somatic recordings were performed using a Multiclamp 700A amplifier (Axon Instruments, Germany) and an upright microscope (Olympus BX51-WI, Olympus, Switzerland). 

The KMeSO_4_-based intracellular solution contained (in mM): 5 Na-ATP, 0.3 Tris-GTP, 14 Tris-phosphocreatine, 20 HEPES, and either 125 KMeSO_4_, 5 KCl, or 90 KMeSO_4_, 40 KCl; 285 mOsmol and pH 7.35 adjusted by potassiumhydroxide titration. We used borosilicate electrodes for whole-cell patch-clamp recordings (1.5 mm external diameter, 1.17 mm internal diameter) without filament and an open tip resistance of 5-6 MΩ. 

Background fluorescence increases due to dye spillage was avoided by tip-filling the electrode with dye-free solution. In addition, before reaching the cells, positive pressure in the pipettes was kept low in the bath and controlled with a manometer at ~ 5 mbar (Model 840081; Sper Scientific, Scottsdale, AZ). Staining time was determined by measuring the resting fluorescence from the cell body at reduced excitation light intensity (~0.1 % of the laser light). Cell loading with VSD did not cause pharmacological effects [8,11]. After enough dye had diffused into the cell (30 min), pipettes were gently removed by forming outside-out patches. Optical recordings were performed when dendrites were sufficiently filled with VSD (~ 20-30 min after electrode removal). 

### Electrophysiology

If relevant for the experiments, electrical signals were detected at the beginning of the experiments simultaneous to optical signal detection.

For paired recordings, potentially connected interneurons of CA1 stratum radiatum were searched, while pyramidal cells were being filled with the dye. To improve the detection of small unitary inhibitory postsynaptic potentials (uIPSPs), pyramidal cells were patched with intracellular solution containing 40 mM Cl^-^. Hence, the driving force for chloride increased, potentials were larger in amplitude and evoked potentials were positive due to a more depolarized reversal potential for Cl^-^. 

Somatic electrode recordings were acquired at 16 kHz and filtered at 4 kHz by using the Redshirt imaging system or acquired at 20 kHz and filtered at 2 kHz by a separate A/D board (NI USB-6343, National Instruments, Switzerland).

### Optical recordings

Excitation of the VSD was achieved using a 532 nm- 300 mW solid-state laser (model MLL532; CNI, China). Hence, the dye was stimulated at the border of its absorption spectrum and the largest dynamic range in fluorescence could be reached. 

Advantages of laser illumination compared to a conventional xenon arc lamp were described earlier [8]. 

GABA_A_-receptor-mediated uIPSPs and evoked EPSP/IPSP patterns were detected optically at a frame rate of 500 Hz and 6 % of the full laser intensity. Optical signals were captured with a high-speed, 80 x 80 pixels CCD camera (NeuroCCD-SM, RedShirtImaging LLC, China). The fluorescence image of the cell was projected via a 0.2 optical coupler onto the CCD camera. The imaged field in our measurements was ~125 μm x 125 μm. The excitation light was directed to the preparation using a 570 nm dichroic mirror and a water immersion objective (Olympus 60x/1.1 NA, Olympus, Switzerland). The emission light was filtered with a 610 nm long-pass filter. 

Optical signals were averaged over 10-20 pixels along compartments of the stained cells, at a pixel size of 1.56 µm x 1.56 µm. To improve the signal-to-noise ratio, averages of 3-12 trials were taken.

For analysis, dendritic sub-compartments (20-50 pixels in size) were chosen by comparing VSD fluorescence images with two-photon reconstructions of the neurons. Direct comparison of DF/F signals between different cellular compartments have to take into account the possibly uneven dye distribution. We have therefore used the ratio between de- and hyperpolarizing signals as our main measurement – these ratios are not affected by dye inhomogeneity. 

### Stimulation and Pharmacology

Extracellular stimulation was performed by using borosilicate patch pipettes filled with ACSF. Hydraulic manipulators (Narishige) were used to place pipettes. Feedforward inhibition was evoked by stimulation of Schaffer collaterals in stratum radiatum between CA3 and CA1. They innervate different types of interneurons located in stratum pyramidale and *radiatum* [6].

Stimulation pulses were 0.1 ms of duration and strength varied between 20 to 60 μA. Pulses were delivered by an IS4 stimulator (SC-Devices, Switzerland) and triggered by stimulation protocols written in IGOR Pro software (Wave Metrics, USA).

GABA_A_R-mediated potentials were tested by bath application of the competitive receptor antagonist bicuculline (20 μM).

Direct stimulation of interneurons in the stratum radiatum was avoided by low current injection and careful placement of the stimulus electrode. Control experiments to test for feedforward inhibition were performed by applying 2,3-dioxo-6-nitro-1,2,3,4-tetrahydrobenzo[f]quinoxaline -7-sulfonamide disodium salt (NBQX, 20 μM),a specific α-amino-3-hydroxy-5-methyl-4-isoxazolepropionic acid receptor (AMPAR) antagonist, to block signals from Schaffer collaterals to interneurons and pyramidal cells.

### Anatomical reconstruction and analysis

Anatomical reconstruction of neurons was carried out from a stack of two-photon excitation fluorescence images obtained using a tunable, modelocked titan-sapphire laser (MaiTai HP, Spectra Physics) set to 880 nm and a laser scanning system (FV300, Olympus) with a high-aperture 20x water-immersion lens (Olympus LUMPLAN 20x).

Optical signals were analyzed as fractional changes of fluorescence (ΔF/F). Optical and electrophysiological recordings were analyzed with dedicated software written in MATLAB (The MathWorks). Optical signals were corrected for the bleach fraction. Stimulation artefact was cleared for clarify.

Statistics were calculated in Excel (Microsoft Office 2010) and averages are presented as the mean ± standard error of the mean (SEM). 

## Results

We first tested the suitability of VSD imaging to study feedforward inhibition by comparing classical electrode recordings with VSD imaging. 

**Figure 1:**
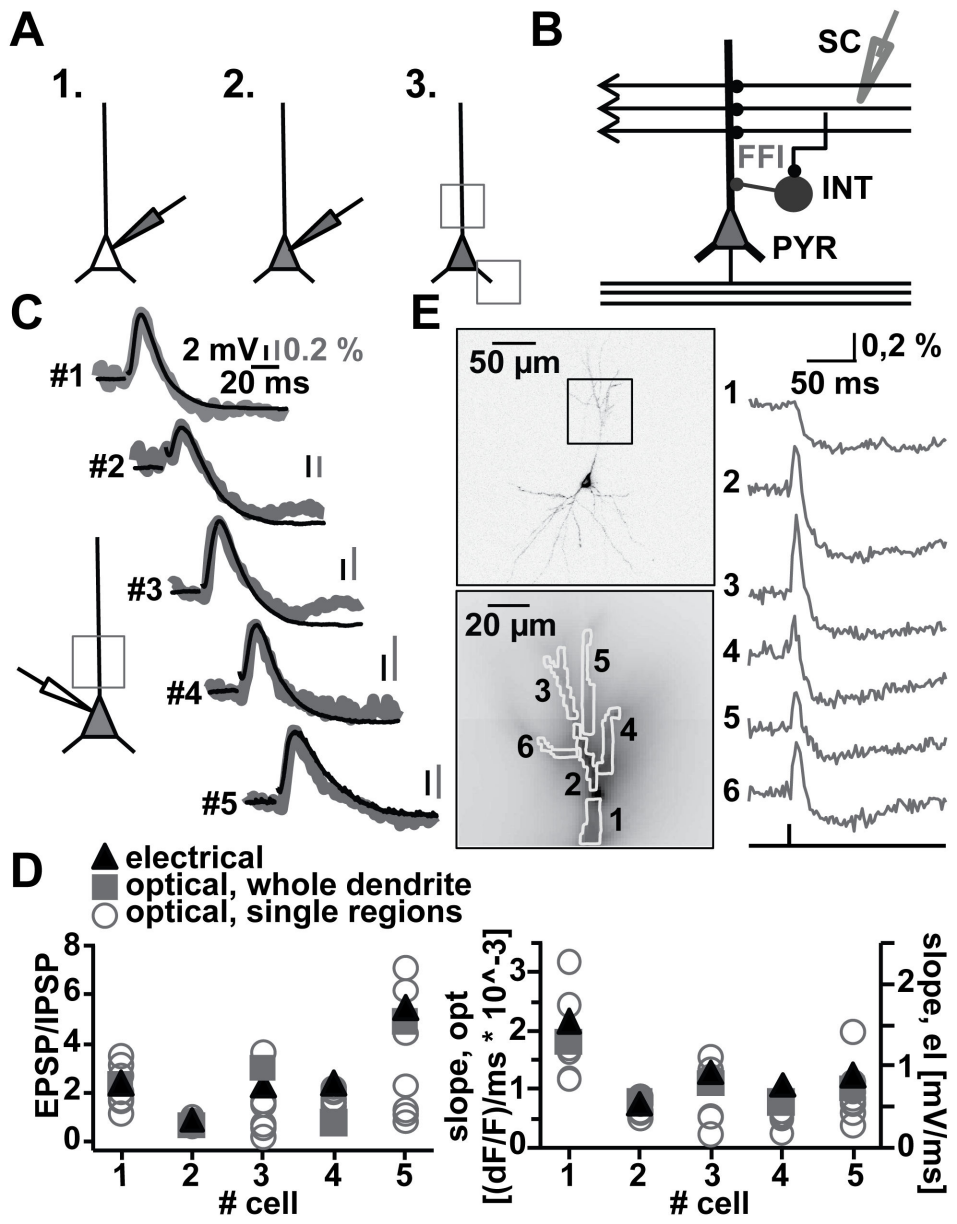
Measuring feedforward inhibition by VSD-imaging. A. Schematic configuration of staining and experimental procedure. Left: whole-cell patch configuration. Middle: filling of CA1 pyramidal cell during patch stage. Right: optical recordings in non-patch stage. Rectangles represent examples of recording sites in filled cells. B. Schematic illustration of stimulation location. Schaffer collaterals (SC) stimulation to activate feedforward inhibition (FFI). INT, interneuron; PYR, CA1 pyramidal cell C. Simultaneous electrical (black trace) and optical (grey trace) recordings of SC evoked EPSP, IPSP patterns in five example cells. Schematic illustration shows electrical and optical recording configuration. D. Scatter plots derived from electrical data and optical data of single regions and averages over the whole dendrite. E. Example of high resolution optical recording after SC stimulation. Left top: two-photon reconstruction of imaged cell; rectangle shows imaged region at the apical dendrite. Left bottom: ROI (1-6). Right: optical dendritic recordings of EPSP-IPSP patterns in the marked ROI (average of 10 trials).

The staining procedure was described in more detail in an earlier publication [8] and is schematically shown in Figure 1 A.

First, we performed whole-cell patch recordings to load hippocampal CA1 pyramidal cells with the VSD JPW1114. We did not observe a significant change in either the amplitude or the time course of SC stimulation-evoked synaptic signals during dye filling (Figure S1). 

After cells were filled adequately, pipettes were slowly removed from the cell somata. The field of view of the camera allowed the optical recording of membrane potential distributions in several regions of interest covering multiple dendritic branches (rectangles, Figure 1 A). 

Feedforward inhibition could be initiated in CA1 pyramidal cells by extracellular stimulation of projecting SC in stratum radiatum of the hippocampus (Figure 1 B). Excitatory potentials initiated by SC synapses projecting to CA1 neurons were followed by a disynaptically-induced hyperpolarisation (Figure 1 C). In a subset of cells, whole-cell patch-clamp recordings were established by somatic re-patching. As shown for five examples in Figure 1 C, both the somatic electrode recording and the average VSD imaging trace show the same de- and hyperpolarizing pattern. The VSD imaging signal over the whole dendritic field and the somatic recordings produced the same EPSP/IPSP ratio and the same slope of EPSP depolarization (Figure 1D solid symbols, Table S1).The correlation coefficient between the two signals was >0.9 for each of these experiments (Figure S2), indicating that feedforward inhibition can be faithfully recorded by VSD imaging. 

Looking at the voltage transients in different dendritic branches and segments revealed significant inhomogeneity in EPSP/IPSP ratio and slope of EPSP depolarization between the different compartments (Figure 1 D open symbols, Table S1), indicating that the average recording is an insufficient predictor for the signal in individual compartments. While an inhibitory component could be observed in all imaged regions of interest (ROI), the amount of depolarization varied considerably (Figure 1 E, ROI 1-6), and could be completely absent (ROI 1). Thus, while the average voltage transient revealed through imaging is similar to the transient observed in somatic whole-cell patch-clamp recordings, individual dendritic branches and segments contribute very differently to this signal (Figure 1 D, E). 

The large variability in the excitation-inhibition ratio suggests an uneven and non-overlapping distribution of activated excitatory and inhibitory synapses. 

**Figure 2:**
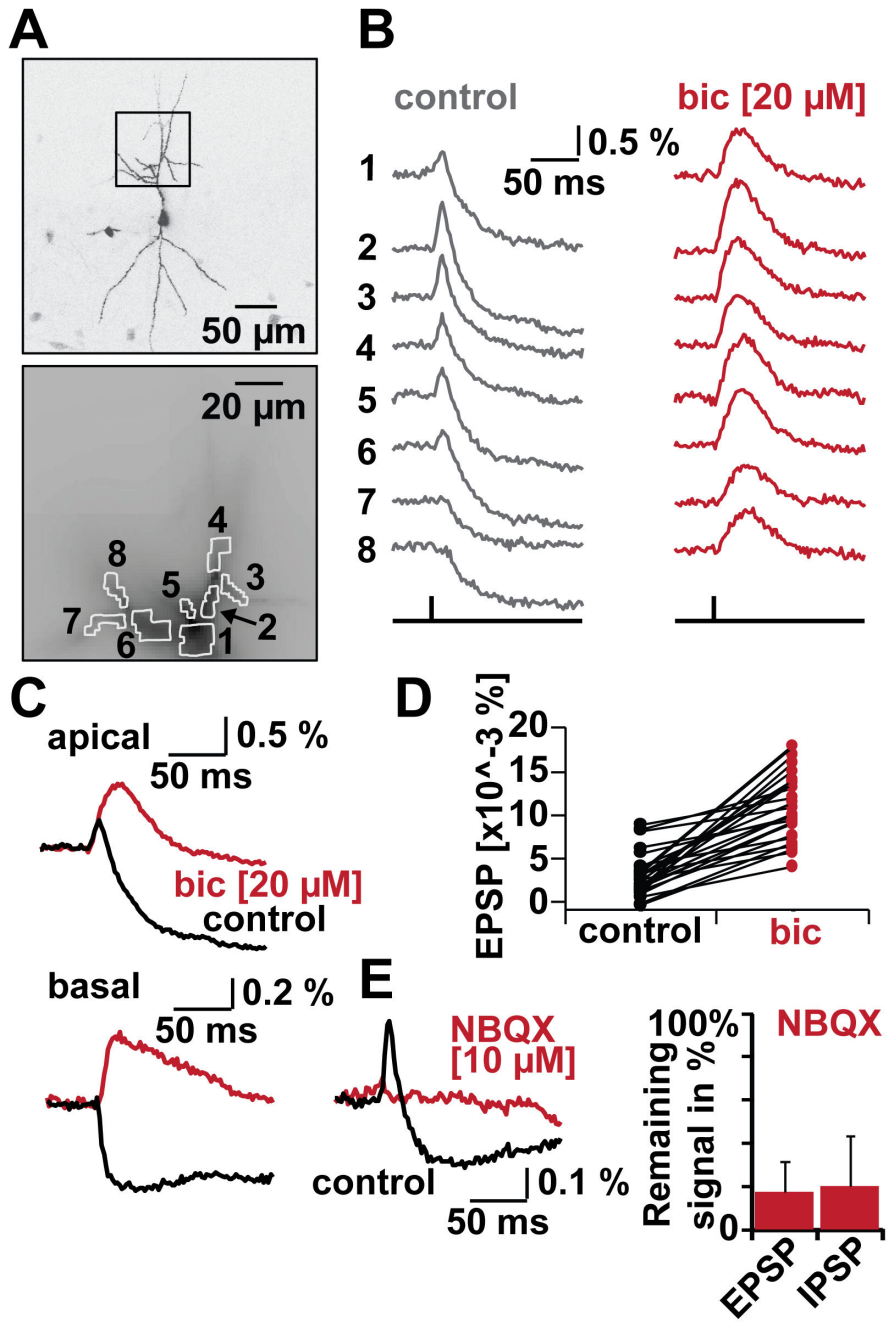
Shaping of dendritic potentials by GABAergic inhibition. A. Top: two-photon reconstruction of imaged cell. Rectangle indicates recording site in apical dendrite. Bottom: ROI (1-8). Averaged pixels per region ~20. B. Bicuculline (bic, 20 μM) effect on SC evoked potentials in apical dendritic ROI. Region assignment as in A. Traces are averages of 6 trials. Left: control conditions. Right: bic conditions. See also supportive Movie S1. C. Overlay of control (black) and bic (red) traces. Top: responses in apical dendrite (average of 6 trials). Bottom: responses in basal dendrites (average of 5 trials). D. Effects on EPSPs under control conditions (black) to bic substitution (red). N (regions)=26 out of N (cells)=4. E. Left: Test for disynaptic inhibition with bath-application of NBQX (10 μM) to block glutamate-mediated excitation; control (black) and NBQX (red), traces are averages of 11 trials for control, respectively 7 trials for NBQX. Right: NBQX reduced EPSPs to 17.2±14.5 %. IPSPs were reduced to 19.7±23.5 % (N=6 cells).

How much are the transients shaped simply by the distribution of activated synapses and how much does the interaction of the opposing signals contribute? To measure the influence of GABA_A_R-mediated inhibition in shaping dendritic voltage transients, we applied the specific blocker bicuculline (Figure 2), while cells were imaged as described before. 

Under control conditions, optical recordings in dendritic subregions (ROI 1-8) along the apical dendrite revealed an inhomogeneous EPSP/IPSP distribution (Figure 2 B, left). Note the complete absence of EPSPs in two side branches (ROI 7 and 8). Bath application of bicuculline (20 μM) completely suppressed IPSPs in all regions imaged (Figure 2 B, right). Moreover, robust depolarizing potentials throughout the dendrite were observed in the absence of GABA_A_R mediated inhibition – notably in areas in which no EPSP was visible before (ROI 7 and 8). A robust increase in depolarization was observed in all 26 ROIs in four cells imaged (Figure 2D), which was accompanied by a loss of the hyperpolarizing signal (data not shown). Comparison of the signals from the different ROIs in a given cell showed a clear increase in their correlation after the addition of bicuculline in all four cells (Figure S3, Table S2). This indicates that GABAergic signals substantially contribute to the variability of the dendritic signals. Animated data is available in the movie (Movie S1). In accordance with previous electrode recordings [3] the excitatory transients also lasted substantially longer. This was particularly visible in average scans from apical and basal dendrites (Figure 2 C). 

All synaptic signals evoked by SC stimulation were sensitive to the addition of the AMPA receptor antagonist NBQX (10 μM; Figure 2 E) to the perfusate. EPSPs were reduced to 17.2 ± 14.5 % and IPSPs to 19 ± 23.5 % (N=6 cells) (Figure 2 E, right). Thus almost all of the inhibitory synaptic signals were originating from interneurons indirectly activated by SCs, representing feedforward inhibition.

**Figure 3:**
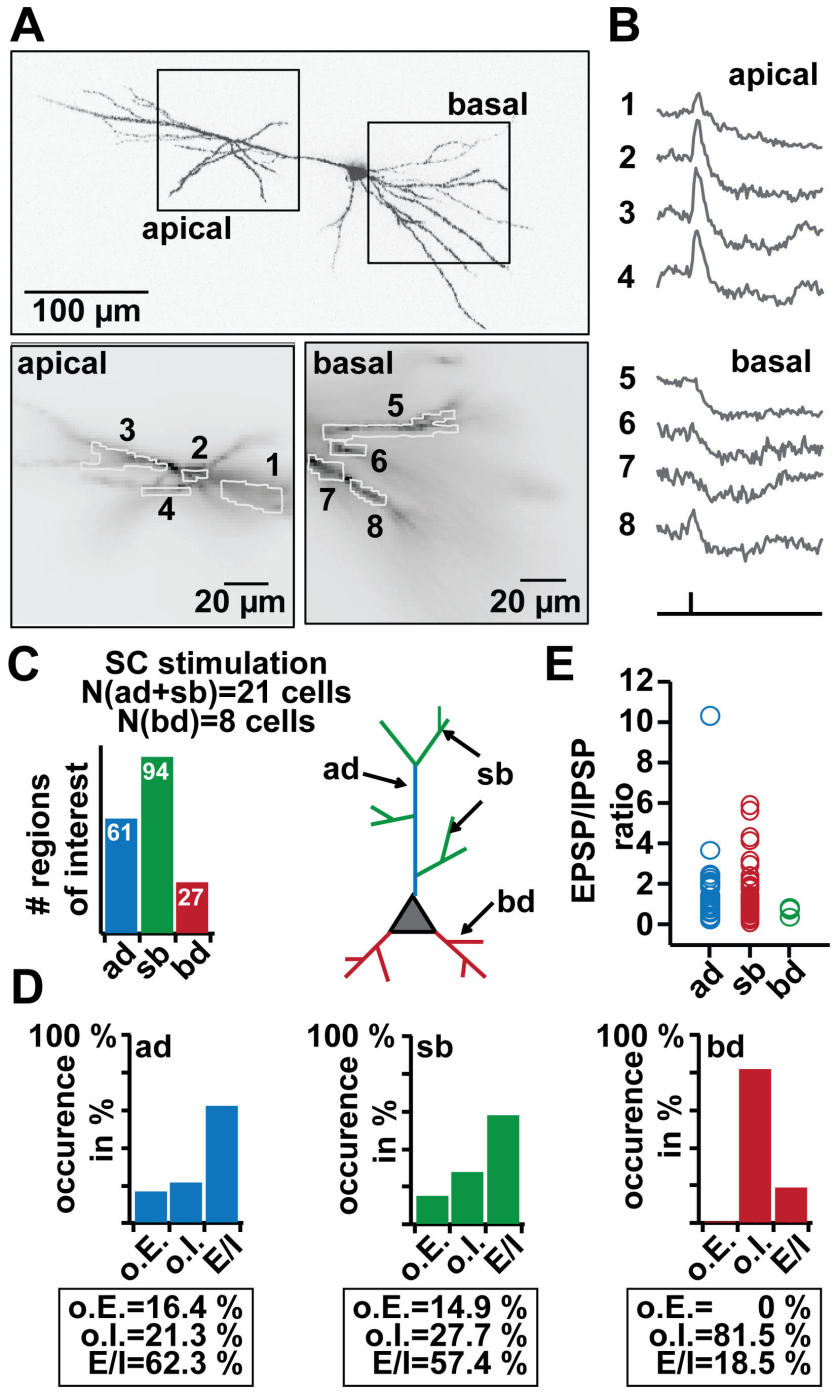
EPSP-IPSP patterns in distinct dendritic branches. A. Top: two-photon reconstruction of imaged cell. Rectangles show recording site at the apical and basal dendrite. Bottom: ROI in apical (1-4, left) and basal (5-8, right) dendrites. Sum of averaged pixels per region ~20-50. B. Optical recordings of EPSP-IPSP patterns in apical (ROI 1-4) and basal (ROI 5-8) dendrites evoked by SC stimulation. ROI as in A (recordings were averages of 4-10 trials). See also supportive Movie S2. C. Classification of measured dendritic subcompartments (20-50 pixels) in SC experiments. ap = apical dendrite (blue), sb = side branch (green), bd = basal dendrites (red). Total of cells N = 21 (thereof eight also for experiments in the basal dendrites). Left: plot of total analysed ROIs; Right: schematic illustration of CA1 pyramidal cell, colour code shows subdivision of the dendrites in ap, sb and bd. D. Frequency of occurrence plots for only EPSPs (o.E.), only IPSPs (o.I.) or EPSP-IPSP (E/I) sequences in ad, sb or bd ROIs. 100 % represents the total amount of regions measured for ad, sb or bd. E. Distribution of relative amplitudes for EPSP-IPSP sequences in ap, sb and bd.

Dendritic propagation of depolarizing signals within the dendritic tree is actively controlled by feedforward inhibition in CA1 pyramidal neurons. To quantify these effects we analysed 182 of ROIs in 21 imaged cells. We subdivided the dendritic tree into broad anatomical categories, apical main trunk (ad, blue), apical side branches (sb, green) and expanded our analysis in particular to basal dendrites (bd, red) (Figure 3). Imaging dendritic responses to SC stimulation revealed the familiar inhomogeneous de- and hyperpolarizing pattern in ad and sb segments (Figure 3. B, ROI 1-4), while bd segments showed nearly uniform hyperpolarization (Figure 3. B, ROI 5-8). See also supplemental material for animated data (M2). Quantitatively, the majority of bd compartments (22 of 27 imaged) showed only an inhibitory response, while a minority (13 of 61 imaged) of ad and (26 of 94) of sb segments exhibited solely an IPSP (Fischer test p<0.01) (Figure 3 D). For the compartments with EPSP-IPSP sequences we observed a large variability in their relative strength. The average EPSP-IPSP ratio was 1.53 (CV 1.08, N=38) for ad, 1.75 (CV 0.71, N=54) for sb, but only 0.69 (CV 0.28, N=5) for bd segments (Figure 3 E). 

Depolarization occurs to a variable degree and only in a subset of dendritic compartments, it is notably absent from many basal dendrites after SC stimulation. Are these patterns static or can they be altered by synaptic plasticity?

**Figure 4:**
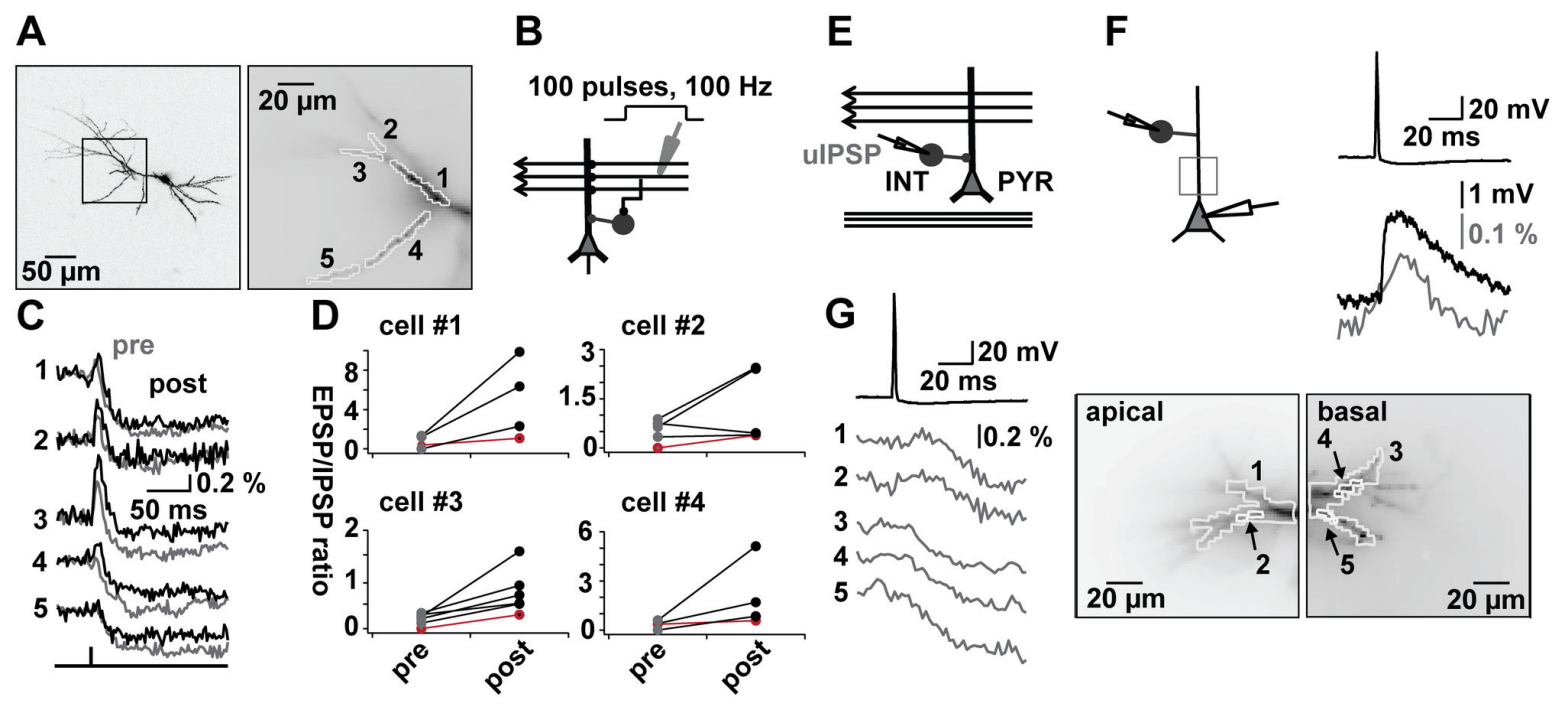
Plasticity of EPSP-IPSP patterns. A. Left: two-photon reconstruction of pyramidal cell. Rectangle represents recording site. Right: ROI in apical dendrite (1-5). Averaged pixels per ROI ~20-50. B. Schematic illustration of plasticity protocol. Application of tetanus in SC (100 pulses, 100 Hz). C. Trace-overlay of evoked potentials before (pre, grey) and after (post, black) the tetanic stimulation for one example cell. Region assignment as in A. Signals are averages of 8 for pre, respectively 4 for post tetanic trials. D. Plots of EPSP-IPSP ratios pre- and post-tetanus for different ROIs in apical dendrites of 4 cells. Red traces indicates the most proximal dendritic compartment. Size of ROIs ~20-50 pixels. E. Schematic configuration illustration for synaptically connected cell pair recording between INT of stratum radiatum and PYR to measure unitary IPSPs (uIPSPs). F. Simultaneous somatic electrical (black bottom trace) and dendritic optical ΔF/F (grey trace) recording of an uIPSP in response to a spike in presynaptic interneuron (top trace). Signals are averages of 3 trials. G. Example of optical recordings of uIPSPs at higher spatial resolution. Left: top trace spike in presynaptic interneuron, optical traces of evoked uIPSP in ROI (average of 3 trials). Region assignment on the right. Right: fluorescence image of apical (region 1, 2) and basal (region 3-5) dendritic ROIs of sample CA1 pyramidal cell. ROI 1 and 3 are spatial averages over the whole dendrite in focus.

To study this issue, we applied simple tetanic stimuli in the SC pathway (100 pulses by 100 Hz; Figure 4 B), commonly used to induce synaptic plasticity. Optical recordings in CA1 pyramidal cell dendrites were taken shortly before and after the tetanic stimulus in several ROIs of four different neurons (N=29 ROIs). EPSPs and IPSPs showed stable baselines within a time interval of 150-250 s before tetanisation (p(EPSPs) = 0.175; p(IPSPs ) = 0.347; Figure S4). In Figure 4 A ROIs (right side) for one example neuron (left side) are illustrated. An overlay of pre (grey traces) and post (black traces) tetanic optical recordings (Figure 4 C) showed a change in the EPSP-IPSP pattern in several, but not all subcompartments. The changes were more pronounced in distal compared to proximal compartments and resulted in an increase in the EPSP-IPSP ratio. Such changes were found in all four cells tested (Figure 4 D) – with the red line indicating the result from the most proximal compartment imaged in each cell. Overall the tetanic stimulation resulted in a highly significant alteration in the EPSP-IPSP patterns (p=0.0003; Rank-Sum-Test). 

The pronounced inhomogeneity in the dendritic excitation-inhibition patterns is particularly visible for the excitatory side. What is the reason for the more homogeneous inhibitory component? To address this question we investigated the contribution of single interneurons to the inhibitory response in CA1 pyramidal neurons. 

We produced paired recordings between neighbouring GABAergic neurons and dye-filled principal cells (N=7; Figure 4 E). We focused on interneurons located in the stratum radiatum – which have a high probability to receive input from SCs and to contribute to feedforward inhibition [2]. Interneurons were stimulated by eliciting action potentials (black trace, Figure 4 F) through somatic current injection. To enhance the chances of detecting connected pairs we increased the size of GABAergic postsynaptic potentials by dye-loading pyramidal cells with an intracellular solution containing 40 mM chloride. A simultaneous electrical (black, somatic electrode) and optical (grey, average over the apical dendrite) recording of a pair is shown in Figure 4 F (bottom). Retraction of the loading pipette resulted the re-establishment of physiological, hyperpolarizing chloride gradients in the pyramidal cell, as shown previously [8]. We now studied the responses in different apical (ROI 1 and 2) and basal (ROI 3-5) compartments (Figure 4 G) of the target pyramidal cell. We did not detect major inhomogeneities in the dendritic responses in any of the cells imaged. 

## Discussion

We have revealed a significant degree of inhomogeneity in dendritic membrane potential transients in CA1 pyramidal cells after SC stimulation and have shown this to be largely the product of the interaction of excitatory and inhibitory synapses. While NBQX-sensitive feedforward hyperpolarization was detected in almost all dendritic subregions, many dendritic segments, particularly in basal dendrites showed no discernible depolarizing responses. The small fraction of NBQX resistant inhibition cannot explain the profound influence of feedforward inhibition on dendritic membrane potential dynamics, evidenced by the large effect of bicuculline on dendritic excitation. In the absence of GABA_A_R mediated feedforward inhibition, excitatory potentials did spread throughout the dendritic arborization, indicating that these patterns are actively shaped by the network of interneurons surrounding CA1 pyramidal cells [2,12]. Active shaping of spatial membrane potential profiles by feedforward inhibition were also observed by Tominaga et al. [13], who found a selective perisomatic inhibition after SC stimulation. As we clearly show, feedforward inhibition is temporally so tightly coupled to excitation that it reaches a substantial fraction of the dendritic tree before excitatory signals spread there. Thus, processes that rely on dendritic depolarization, such as the opening of voltage-gated conductances or NMDA receptor activation, are restricted to specific dendritic regions. Antagonists of GABA_A_ receptors have long been shown to dramatically facilitate long-term potentiation [14]. As we now confirm, they not only affect the overall excitability of the postsynaptic neurons, but profoundly alter synaptic integration - facilitating the interaction between synapses on distant dendritic branches. 

The control of excitation in time and space by GABAergic inhibition is crucial in shaping the propagation of information throughout the central nervous system. This is particularly evident in sensory processing. In the spatial domain, GABAergic neurons reduce the activity of nearby pyramidal cells, sharpening and controlling their excitatory profiles. Such activity has been observed in several areas, e.g. the whisker barrel cortex or the visual system [15–17]. Blocking GABA_A_R mediated inhibition broadens the tuning curves of pyramidal neurons in olfactory [18], auditory [19] and visual cortex [20]. In the temporal domain feedforward inhibition in the hippocampus [3] and cortex [21] was shown to shorten and thus control the integration time window for excitatory synaptic input. 

Both excitatory and inhibitory inputs to CA1 pyramidal cells show layer-specific topographic organization consistent with the patterns we have observed. SCs axons from CA3 neurons close to the hilus contact chiefly apical dendrites and their branches, while CA3 pyramidal cells close to CA1 send their axons mostly to basal dendrites [5]. The extracellular stimulation of the classical SC pathway at the border of CA3 to CA1 used in our experiments preferentially activates fibers, which terminate in the apical stratum radiatum [22], consistent with the excitatory focus observed in the apical dendrite and the near absence of excitation in basal dendrites. However, as we show, this selective excitatory innervation results in a focused dendritic depolarization only in the presence of feedforward inhibition. 

The precise subtype of the involved interneurons could not be characterized. The strong evidence of compartment-specific innervation by different subtypes of interneurons [6] can explain the selective silencing of dendritic branches. 

We thus postulate a dynamic network of excitatory and inhibitory connections innervating pyramidal neurons such that excitatory inputs are surrounded and controlled by inhibitory synapses. For example, basket cells, which innervate the soma, have been shown to participate in feedforward inhibition [1,23] and are likely to control the spread of apical EPSPs to basal dendrites.

VSD imaging is particularly suited to the study of dendritic signals in the extremely thin basal dendrites. To date, their integrative properties remain elusive due to the difficulties in recording from them with classical electrode techniques. Recently, it has been shown in layer V pyramidal neurons that they exhibit strong synaptic scaling and can generate active signals [24,25]. Given their high input resistance they are particularly susceptible to GABAergic shunting, which may control these active properties [26,27]. 

Paired recordings between individual interneurons and CA1 pyramidal cells showed relatively uniform hyperpolarizing signals in both basal and apical dendrites. The influence of GABA_A_Rs on dendritic EPSPs can therefore be best explained by their shunting effect, which is significantly more localized than the hyperpolarization they cause [23]. Thus, to understand the subcellular signalling of different interneuron subclasses it is important to design experiments that reveal shunting inhibition. 

Dendritic membrane potential patterns by themselves are not fixed, but can be altered by stimuli that induce synaptic plasticity. Interestingly, this plasticity is non-uniform in itself – proximal portions of the apical dendrite showed small changes in the EPSP-IPSP ratio, while distal portions showed a more pronounced increase. The exact locations of the synaptic changes will need further investigation.

Intrinsically, synaptic integration in neurons is controlled in time by the membrane time constant and in space by the dendritic length constants. As has been shown previously for the temporal domain [3] and here for the spatial domain, feedforward inhibition dramatically improves on these limits and allows a much more precise control over both when and where excitatory synaptic signals can interact.

## Supporting Information

Figure S1 **Effect of VSD wash-in on cell physiology.** A. Schematic configuration of VSD-staining during whole-cell patch configuration. Stimulation electrode (stim) was placed close to soma next to the dendrite. Bottom: Time windows in ms of used abbreviations. B. Bar graphs illustrating averaged slopes (left) and amplitudes (right, amp) of extracellular evoked EPSC at 3 different time points measured during voltage clamp configuration at ~-60 mV. Slope: beg=-100.3±25 pA/ms, mid=-89.1±46.6 pA/ms, end=-89.7±61.3 pA/ms; data do not show significant changes, p(beg-mid)=0.42, p(beg-end)=0.59, p(mid-end)=0.94. Amplitude: beg=280.7±59.7 pA, mid=261.5±117 pA, end=251.5±150.4 pA; data do not show significant changes, p(beg-mid)=0.53, p(beg-end)=0.48, p(mid-end)=0.65. N=8. C. Same cells as in C. Process of slopes and amplitudes in single cells over time. D. No significant changes in cell capacity (C, left), resistance (R, middle) and AP amplitude (AP amp, right) of patched cells during VSD wash-in. N=8. Time windows as shown in A.
(TIF)

Figure S2 **Correlation of simultaneous electrical and optical recordings.** Left: Traces of simultaneous electrical and optical recordings of 5 example cells, same as in Figure 1. Right: Correlation plots of electrical and optical data for each cell. Data are highly correlated; correlation coefficient ρ (right side within each plot) > 0.93. Higher noise levels in the optical signal lead to baseline points being lined up horizontally (arrowhead). Schematic inlet shows electrical and optical recording configuration.
(TIF)

Figure S3 **Loss of inhomogeneity by blocking GABA_A_ receptors.** Correlation plots for optical signals from different ROIs within one example cell under control (top, black) and bicuculline (bottom, red) conditions.
Each plot shows the correlation between the optical signals from two different regions. x-axis components originate from region i, with i being the row number of the plot. y-axis components originate from region j, with j being the column number of the plot. For example, plot in row 2, column 3 shows the signal correlation between regions 2 and 3.
Signal amplitude was normalized to [0,1] for all data. Correlation coefficient is invariant under this procedure.
(TIF)

Figure S4 **Test for stability of baseline in plasticity experiments.** DF/F was taken over the whole dendrite in two trace packages (I and II; each was an average of 5 traces; N (cells)=4) within a time interval of 150-250 s before tetanisation. No significant change was observed in the EPSPs (solid, p = 0.175) and the IPSPs (none solid, p = 0.347).
(TIF)

Table S1 **Electrical and optical data for EPSP-IPSP ratios and EPSP slopes.** See plots in Figure 1, D.
(TIF)

Table S2 **Correlation coefficients from example experiment in Figure S3.**
(TIF)

Movie S1 **Effect of bicuculline (SR stimulation).**
(MP4)

Movie S2 **Distribution pattern in apical and basal dendrites (SR stimulation).**
(MP4)
