# Supplementary figures and images for: Hippocampal Feedforward Inhibition Focuses Excitatory Synaptic Signals into Distinct Dendritic Compartments
Source: PLoS One. 2013 Nov 11;8(11):e80984. doi: 10.1371/journal.pone.0080984 (PMC3823620; doi:10.1371/journal.pone.0080984)

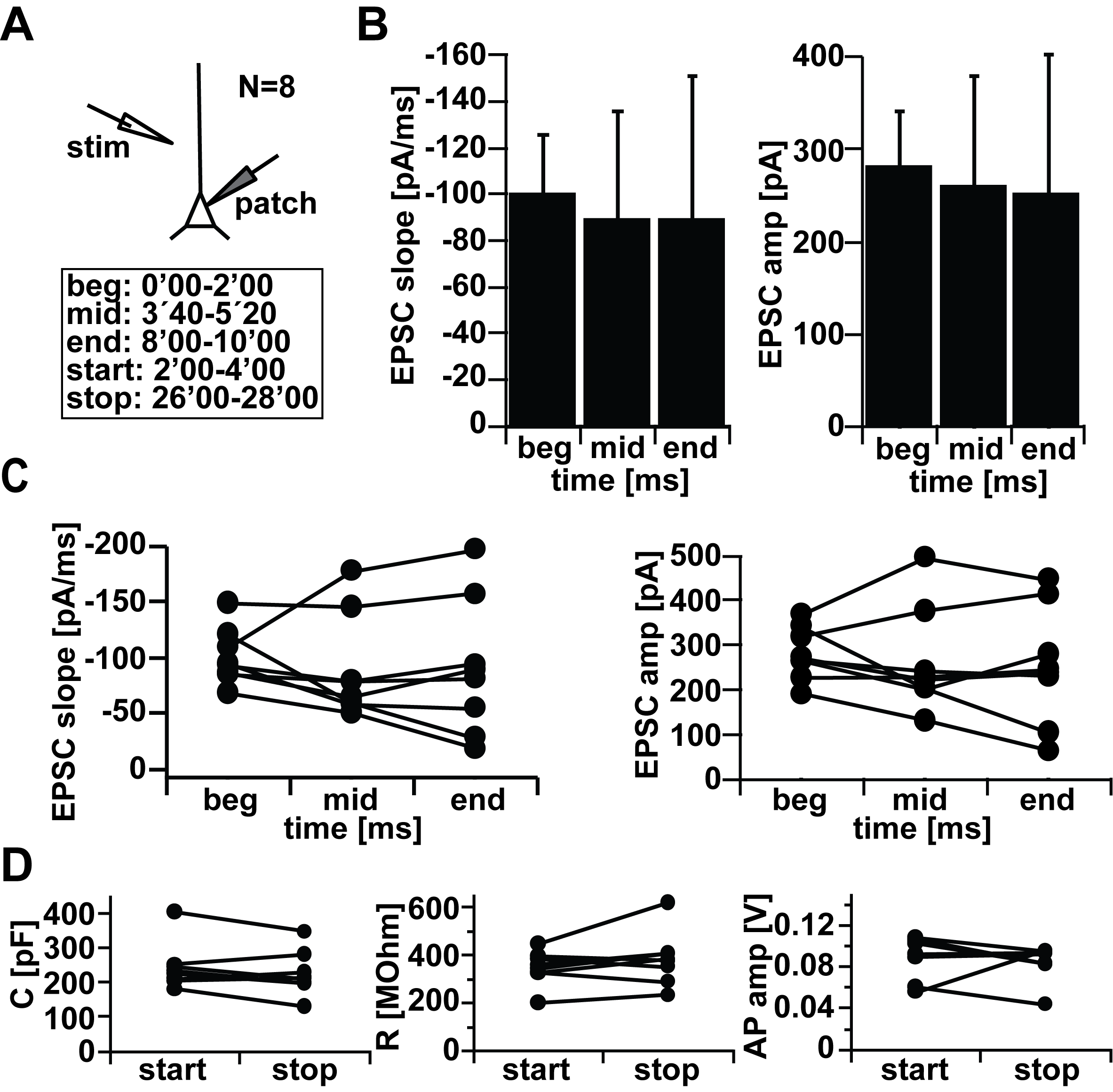

Supplement: Figure S1 — Effect of VSD wash-in on cell physiology. A. Schematic configuration of VSD-staining during whole-cell patch configuration. Stimulation electrode (stim) was placed close to soma next to the dendrite. Bottom: Time windows in ms of used abbreviations. B. Bar graphs illustrating averaged slopes (left) and amplitudes (right, amp) of extracellular evoked EPSC at 3 different time points measured during voltage clamp configuration at ~-60 mV. Slope: beg=-100.3±25 pA/ms, mid=-89.1±46.6 pA/ms, end=-89.7±61.3 pA/ms; data do not show significant changes, p(beg-mid)=0.42, p(beg-end)=0.59, p(mid-end)=0.94. Amplitude: beg=280.7±59.7 pA, mid=261.5±117 pA, end=251.5±150.4 pA; data do not show significant changes, p(beg-mid)=0.53, p(beg-end)=0.48, p(mid-end)=0.65. N=8. C. Same cells as in C. Process of slopes and amplitudes in single cells over time. D. No significant changes in cell capacity (C, left), resistance (R, middle) and AP amplitude (AP amp, right) of patched cells during VSD wash-in. N=8. Time windows as shown in A. (TIF) [file pone.0080984.s001.tif]

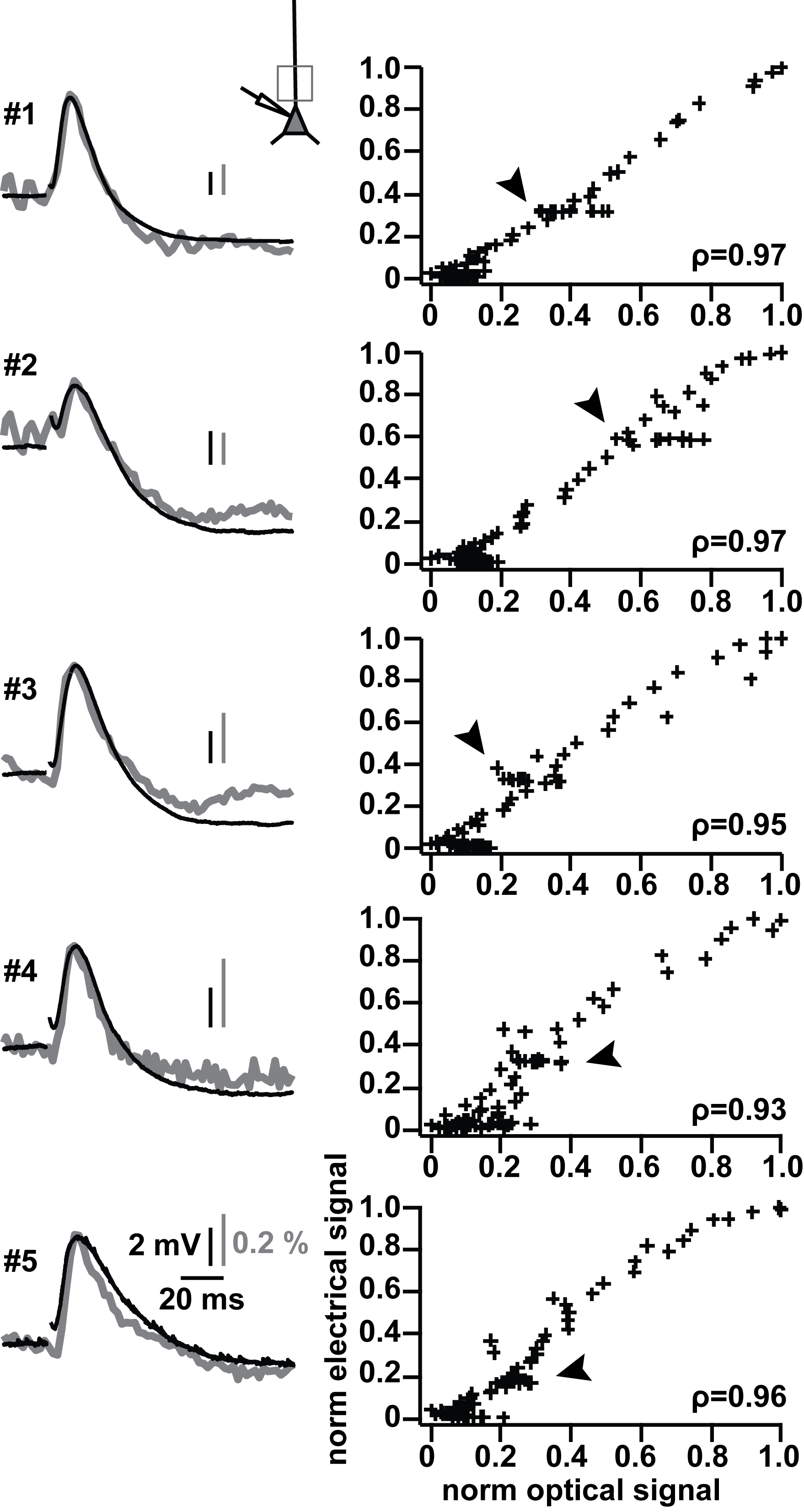

Supplement: Figure S2 — Correlation of simultaneous electrical and optical recordings. Left: Traces of simultaneous electrical and optical recordings of 5 example cells, same as in Figure 1. Right: Correlation plots of electrical and optical data for each cell. Data are highly correlated; correlation coefficient ρ (right side within each plot) > 0.93. Higher noise levels in the optical signal lead to baseline points being lined up horizontally (arrowhead). Schematic inlet shows electrical and optical recording configuration. (TIF) [file pone.0080984.s002.tif]

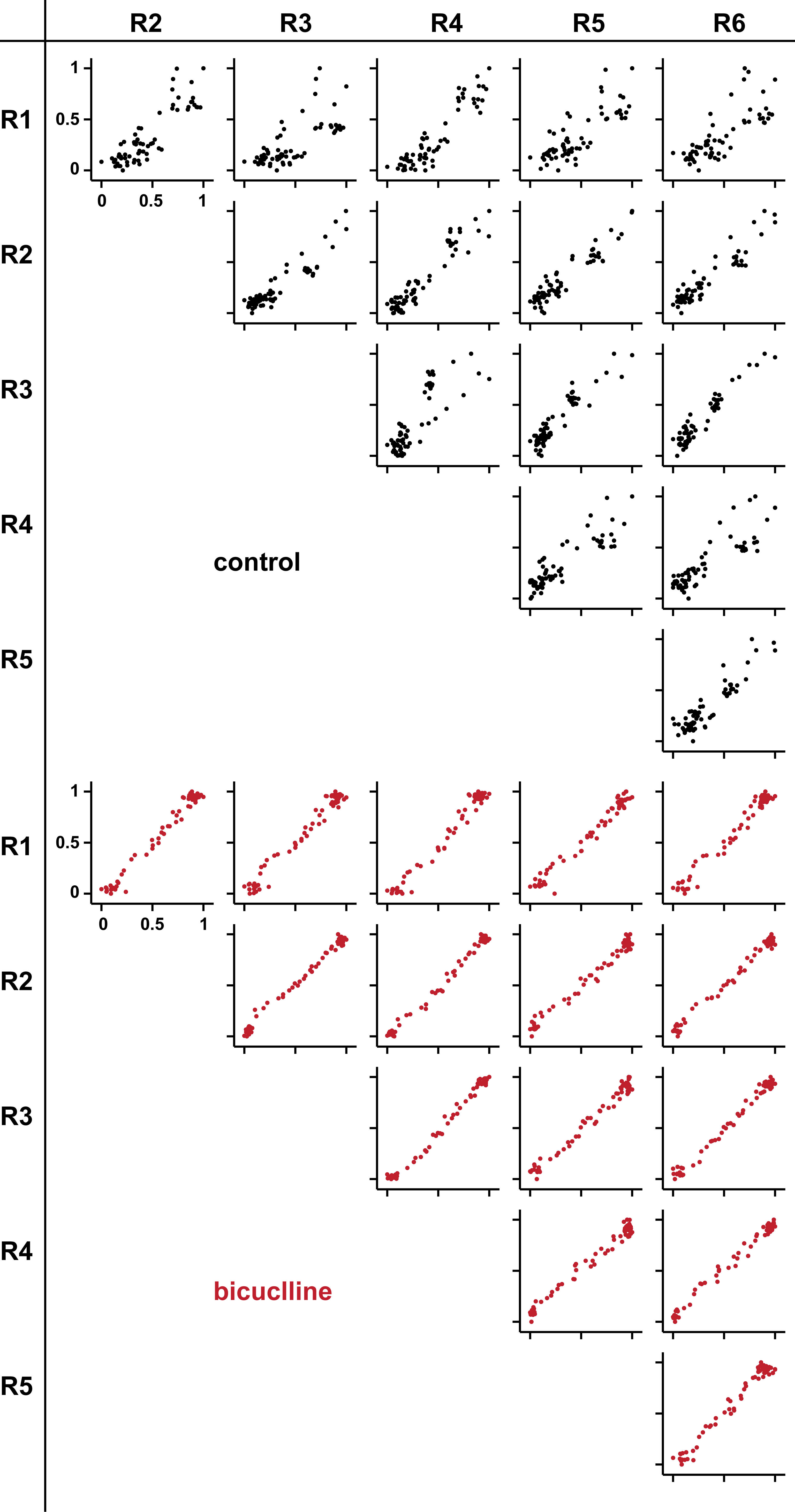

Supplement: Figure S3 — Loss of inhomogeneity by blocking GABAA receptors. Correlation plots for optical signals from different ROIs within one example cell under control (top, black) and bicuculline (bottom, red) conditions. Each plot shows the correlation between the optical signals from two different regions. x-axis components originate from region i, with i being the row number of the plot. y-axis components originate from region j, with j being the column number of the plot. For example, plot in row 2, column 3 shows the signal correlation between regions 2 and 3. Signal amplitude was normalized to [0,1] for all data. Correlation coefficient is invariant under this procedure. (TIF) [file pone.0080984.s003.tif]

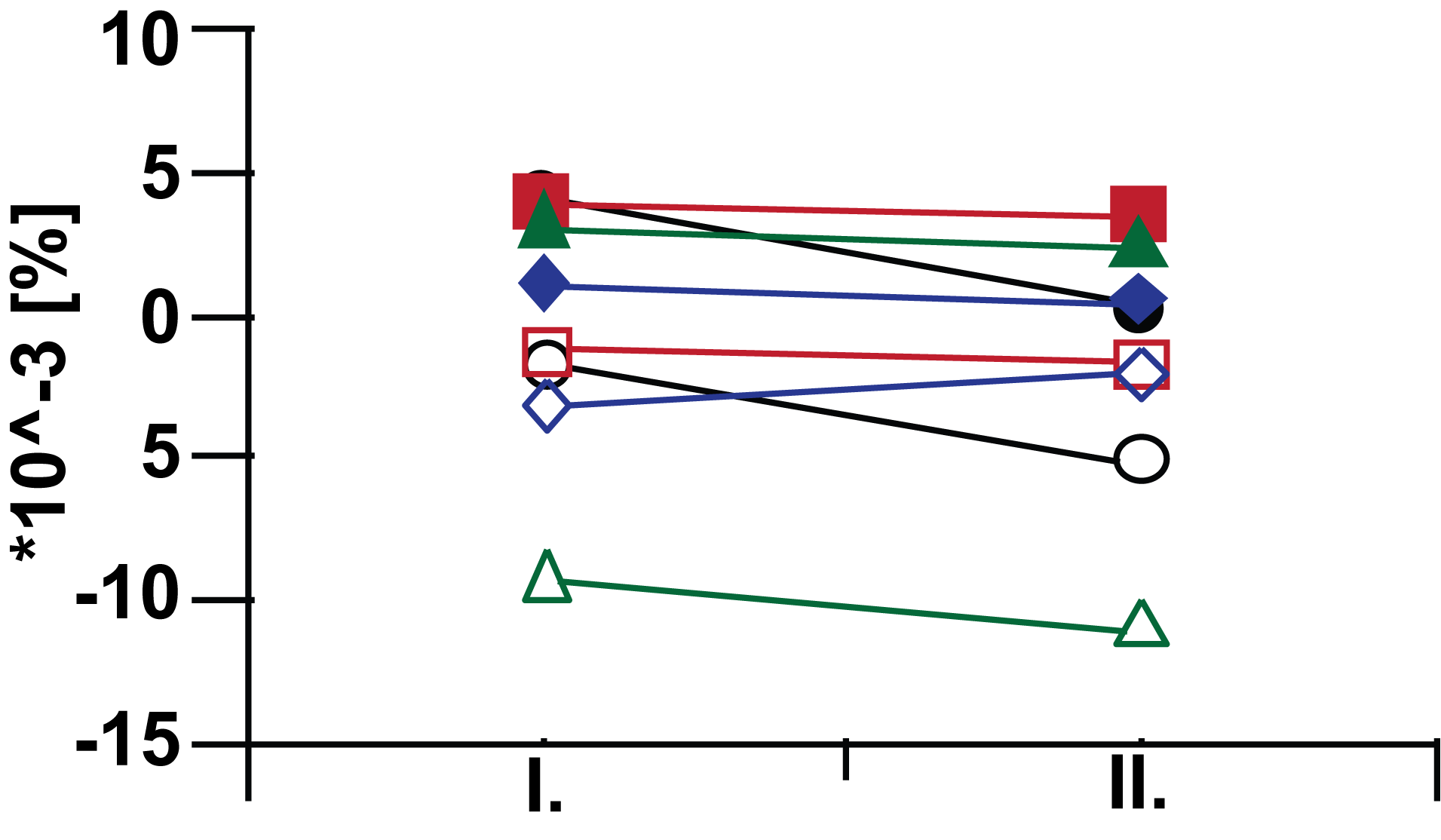

Supplement: Figure S4 — Test for stability of baseline in plasticity experiments. DF/F was taken over the whole dendrite in two trace packages (I and II; each was an average of 5 traces; N (cells)=4) within a time interval of 150-250 s before tetanisation. No significant change was observed in the EPSPs (solid, p = 0.175) and the IPSPs (none solid, p = 0.347). (TIF) [file pone.0080984.s004.tif]

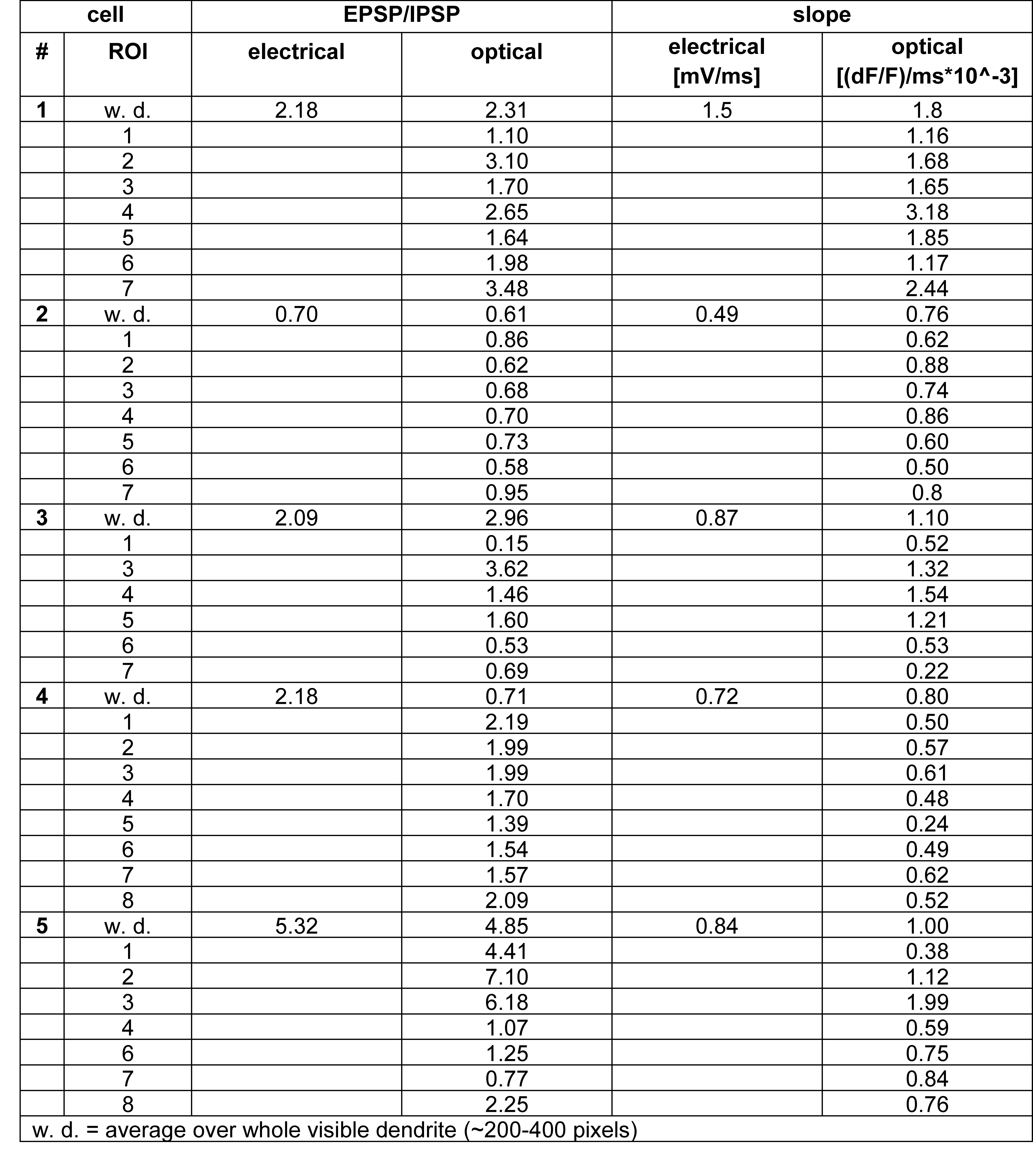

Supplement: Table S1 — Electrical and optical data for EPSP-IPSP ratios and EPSP slopes. See plots in Figure 1, D. (TIF) [file pone.0080984.s005.tif]

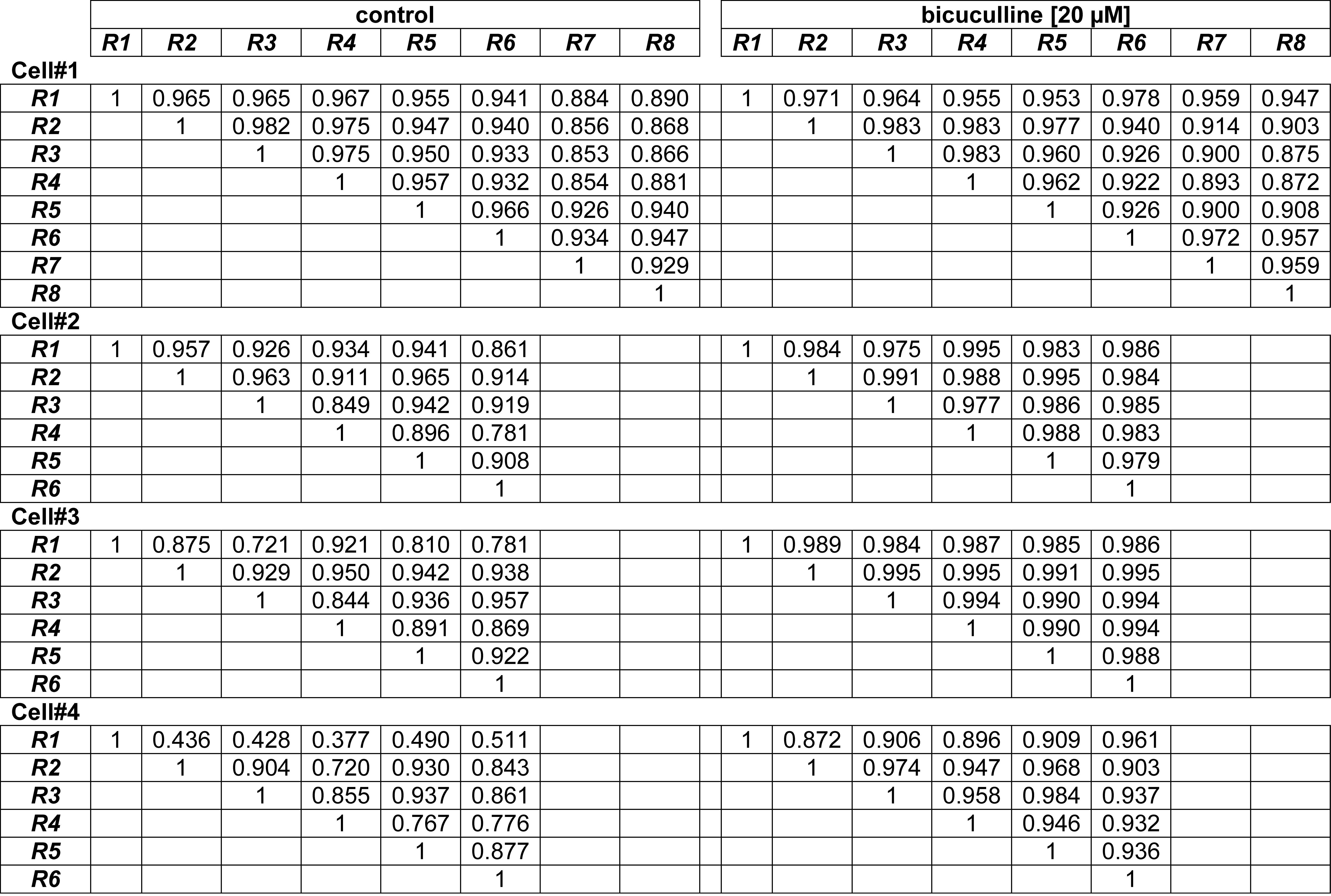

Supplement: Table S2 — Correlation coefficients from example experiment in Figure S3. (TIF) [file pone.0080984.s006.tif]
